# Adverse childhood experiences and intimate partner violence in adulthood among transgender women: exploring the chain mediating role of self-esteem and LGBT minority stress

**DOI:** 10.1080/07853890.2025.2464936

**Published:** 2025-02-12

**Authors:** Lulu Xu, Huifang Xu, Zuxin Wang, Jiani Hu, Yawen Zheng, Fengyi Wang, Ruijie Chang, Ying Wang, Yong Cai

**Affiliations:** ^a^ School of Public Health, Shanghai Jiao Tong University School of Medicine, Shanghai, China; ^b^ Public Health Department, Tongren Hospital, Shanghai Jiao Tong University School of Medicine, Shanghai, China; ^c^ Youth Science and Technology Innovation Studio, Affiliated with Tongren Hospital, School of Medicine, Shanghai Jiao Tong University, Shanghai, China

**Keywords:** Adverse childhood experiences, intimate partner violence in adulthood, self-esteem, minority stress, chain mediator

## Abstract

**Background:**

Transgender women experience a high incidence of intimate partner violence (IPV) in adulthood. The well-documented risk factors contributing to IPV include adverse childhood experiences (ACEs), low self-esteem, and the minority stress experienced by LGBT individuals. The object of the present study was to examine how ACEs influence IPV in adulthood, and to explore the chain mediating function of self-esteem and LGBT minority stress.

**Methods:**

In February 2022, we recruited 264 transgender women through snowball sampling in Shenyang, China. Participants completed a questionnaire assessing background characteristics, ACEs, IPV in adulthood, self‐esteem and LGBT minority stress. Relationships among study variables were examined through variance and correlation analyses. A chain mediation model was tested using PROCESS.

**Results:**

There was a significant correlation among the four variables. ACEs positively predicted IPV in adulthood (*r* = 0.449, *p* < 0.001). The sequential mediation model demonstrated that self-esteem (the estimated effect = 0.0708, 95%CI: 0.0152–0.1327) and minority stress (the estimated effect = 0.0404, 95%CI: 0016–0.0847) had the potential to mediate the connection between ACEs and IPV respectively. Additionally, the combined mediating influence of self-esteem and minority stress (the estimated effect = 0.0298, 95%CI: 0.0105–0.0591) similarly exerted such an effect. In summary, the mediating effect accounts for 34.59% of the overall effect size.

**Conclusion:**

Taken together, the findings underscore the high prevalence of IPV among Chinese transgender women, highlighting the need for additional attention from violence service providers and other healthcare professionals on ACEs, low self-esteem, and minority stress, as these factors may place this population at risk for IPV.

## Introduction

Intimate partner violence (IPV) is characterized as behaviors perpetrated by an intimate partner or a former partner and resulting in physical, sexual, or psychological harm [1], which is a significant public health concern of epidemic proportions, affecting individuals worldwide across various genders and socioeconomic statuses. According to the WHO Report 2018, 27% of women globally had encountered physical or sexual IPV at least once in their lives, with those residing in low-income and middle-income countries being especially susceptible [2]. A recent secondary analysis of population-based surveys revealed that the prevalence of IPV against women aged 15 to 49 years remains notably high, with 37.2% of women experiencing such violence, across 53 low- and middle-income countries spanning from 2000 to 2021 [3]. However, until now, the vast majority of IPV research has been carried out among cisgender females in heterosexual relationships, despite research indicating a higher incidence of IPV (42%∼44.9%) among transgender women [4,5] (i.e. individuals who were assigned male at birth but identifying as female [6]). Several studies, such as systematic reviews and meta-analyses, have indicated that a significant proportion of transgender individuals, ranging from 37.5% to 57%, have experienced IPV. Furthermore, these studies have revealed that transgender individuals are 1.7 times more prone to experiencing any form of IPV, 2.2 times more susceptible to physical IPV, and 2.5 times more inclined to face sexual IPV compared to cisgender individuals [4,5,7,8].

A regularly studied determinant for IPV in adulthood is the occurrence of abuse, neglect, and/or family dysfunction during childhood, collectively termed adverse childhood experiences (ACEs) [9,10]. Particularly, ACEs encompass instances of family dysfunction, such as parental mental illness, substance use, incarceration, and/or witnessing domestic violence. Additionally, ACEs include experiences of maltreatment like physical, sexual, and emotional abuse and neglect, occurring before the age of 18 years [9,11,12]. Prior research consistently revealed that sexual and gender minorities (SGM) frequently report enduring both emotional and physical maltreatment, alongside neglect, before reaching 18 years old, thereby suggesting that these experiences are prevalent within their communities [13,14]. For example, one study highlighted that the three most prevalent ACEs are physical abuse (71.2%), school bullying due to personal identity (67.9%), and exposure to homophobic, biphobic, or transphobic statements made by religious leaders (60.8%) [13]. A biological essentialist view of gender differences and gender ideology may help explain the higher prevalence of ACEs among SGM. This view posits that the unique biological compositions of men and women contribute to their inherent “essences”, leading to mental and behavioral distinctions, shaping masculinity and femininity [15]. These societal beliefs about gender and sexuality, in turn, perpetuate cis heterosexism, which involves the perpetration of violence, discrimination, and unfair treatment against individuals who identify as SGM [16]. Furthermore, Zhu et al. conducted a meta-analysis and illustrated that ACEs were positively linked to IPV victimization [9]. As a unique member of SGM, transgender women may additionally face victimization specific to their transgender identity, encompassing emotional or verbal abuse (such as harassment related to cross-gender appearances or behaviors), and threats (including threats to disclose one’s gender identity). For example, a study involving Black men who are sexually minoritized and transgender women revealed that they experience a high incidence of ACEs; 34% reported having four or more ACE domains [17]. These forms of victimization can exacerbate the adverse consequences of their marginalized status [18]. Nevertheless, there is a deficiency in current research concerning ACEs and their correlation with IPV specifically among transgender women.

Self-esteem is a trait that persists over substantial periods of the lifespan in individuals, which has been linked to childhood factors and intimate relationship violence in previous studies [19,20]. A prospective longitudinal study conducted on a nationally representative sample in the United States has uncovered that self-esteem during early adulthood can be predicted based on various childhood factors, encompassing the quality of the home environment [20]. Attachment theory [21–23] posits that a child’s establishment of a secure attachment to the caregiver results in the formation of a positive internal working model of the self. This model represents the belief that the self is valuable and deserves affection and care from significant others. That is, secure attachment to caregivers could foster young children’s self-esteem. Therefore, if significant others fail to provide children with a responsible, reliable, and fitting parenting environment, children will not recognize their worth and value, potentially leading to the development of low self-esteem that persists into adulthood [24]. In intimate relationships, individuals who suffer from low self-esteem tend to respond to rejection by engaging in more negative self-appraisals, assigning greater blame to themselves, and exhibiting heightened cortisol reactivity [25]. People with low self-esteem tend to be more victimized as they probably lead a life without claims and full of recessions, allowing other people to act arbitrarily against them [26] and may attempt self-protection, conversely resulting in derogation of their romantic partner who is perceived as a threat, finally leading to more physical violence [27]. Conversely, those individuals who possess a higher level of self-esteem demonstrate a more profound aptitude for engaging in personal acceptance in intimate relationships and are less likely to construe behaviors as socially menacing [28]. However, the internal mechanism of self-esteem has been lacking in transgender women populations. Therefore, this study was designed to explore the potential mediating role of self-esteem in the relationship, if any, between ACEs and IPV among transgender women.

In addition to self-esteem, minority stress is another significant variable among transgender women. Minority stress pertains to the additional stress experienced by individuals who face stigma due to their minority status [29]. An investigation was conducted to ascertain whether ACEs predispose individuals to encounter stressful situations in adulthood, which subsequently triggers the emergence of IPV within 231 ethnically diverse newlywed couples inhabiting low-income neighborhoods. The results revealed that for husbands, ACEs were linked to IPV through the mediating factor of stress, whereas no such mediating influence of stress was discernible in the case of wives [30]. The conclusions about husbands align with the premise that a greater number of adversities faced early in life may lead to increased stress levels in adulthood. Consequently, this may weaken their ability to effectively handle issues or disagreements, ­ultimately predisposing them to encounter IPV. Alternatively, the discoveries concerning wives are congruent with social learning theories, indicating that wives’ involvement in IPV might initially stem from modeling experiences during childhood and may later be perpetuated through reinforcement behaviors [31]. As a unique kind of stress, researchers proposed that minority stress including interpersonal victimization, internalized stigma, and rejection experienced by sexual minorities enhances stress levels, ultimately possibly contributing to the development of mental and behavioral difficulties [32,33]. The ways in which minority stress is related to ACEs and IPV remain unknown. It is particularly difficult to find studies that identify minority stress that mediates the relationship between ACEs and IPV specifically among transgender women. We are keenly interested in exploring whether analogous results would be discernible in transgender women, paralleling the discoveries made in husbands or wives in previous research [30]. Therefore, it is necessary to verify the mediation role of minority stress between transgender women’s ACEs and IPV.

Additionally, prior research has suggested that personality traits can serve as the underlying foundation for variations in individuals’ biological reactions to stress [34]. For illustration, when confronted with stressful circumstances, those with diminished self-esteem are inclined to show a heightened negative correlation in their cortisol and adrenocorticotropic hormone (ACTH) reactions. Both of these hormones are secreted by the body in response to stress, suggesting that the lower one’s self-esteem, the greater the perceived stress [35]. Cortisol and catecholamines, particularly norepinephrine and epinephrine, serve as dependable markers of stress responsiveness. They operate within the hypothalamic-pituitary-adrenal (HPA) axis and the sympathetic-adrenal-medullary (SAM) system. These systems are crucial in orchestrating the body’s physiological responses to stress [36]. According to existing research, a sequential relationship exists between self-esteem and minority stress, implying that an individual’s self-esteem may significantly influence their experience of minority stress. Accordingly, we wonder if ACEs affect IPV through mediating the expression of self-esteem and minority stress.

This study aimed to identify the levels of ACEs and IPV exhibited by transgender women and to examine how ACEs influence IPV in adulthood, and investigate the mediating role of self-esteem and LGBT minority stress. Drawing from previous studies, we hypothesized the following: (1) ACEs directly predict IPV; (2) self-esteem mediates the relationship between ACEs and IPV; (3) minority stress serves as a mediator between ACEs and IPV; (4) self-esteem and minority stress jointly mediate the relationship between ACEs and IPV in the chain mediation model.

## Methods

### Participants

Two hundred and sixty-four Chinese transgender women were recruited in Shenyang, China, from February to December 2022. The inclusion criteria were as follows: transgender women aged 18 or older, residing and employed in Shenyang, who self-identified as such, and who expressed a willingness to participate in the study after being fully informed about its objectives and assured of confidentiality. Individuals who self-reported having serious mental illnesses, intellectual disabilities, or an inability to fully participate in the survey were excluded from the study. Participants were provided with clear information that assured them of anonymity and informed them that they could withdraw from the survey at any point without any consequences. The age of the participants varied from 18 to 53 years, with an average age of 29.99 years. Demographic information is presented in Table 1.

**Table 1.** Characteristics of transgender women and IPV in China (*N* = 264).

Variables	IPV^a^	IPV^b^		
	Yes/*N* (%)	Mean (*SD*)	*F*-value	*P*-value
**Age** (mean±*SD*)	264(29.99 ± 6.38)			
**Sexual orientation *n* (%)**			2.085	0.103
Heterosexuality	52(23.3%)	9.61(4.24)		
Homosexuality	112(50.2%)	10.71(3.37)		
Bisexuality	47(21.1%)	10.27(3.51)		
Others	12(5.4%)	8.94(3.30)		
**Education level *n* (%)**			0.822	0.483
Primary school and below	12(5.4%)	9.56(3.16)		
Middle school	51(22.9%)	10.26(4.31)		
High school/vocational school	91(40.8%)	10.62(3.51)		
College degree or above	69(30.9%)	9.88(3.35)		
**Marital status *n* (%)**			2.376	0.070
Married	19(8.5%)	9.13(3.82)		
Unmarried	171(76.7%)	10.16(3.69)		
Divorced	31(13.9%)	11.54(3.08)		
Widowhood	2(0.9%)	9.00(3.46)		
**Monthly income *n* (%)**			6.160	**<0.001**
RMB 3000 and below	58(26.0%)	8.96(3.56)		
RMB 3001–6000	71(31.8%)	10.18(3.66)		
RMB 6001–12000	69(30.9%)	11.32(3.28)		
RMB 12001 and above	25(11.2%)	11.12(3.92)		
**Time of local residence *n* (%)**			2.245	0.084
Local residents	97(43.5%)	9.88(3.93)		
1 year and below	24(10.8%)	10.77(3.67)		
1–5 years	59(26.5%)	11.12(2.99)		
5 years and above	43(19.3%)	9.70(3.58)		
**Working condition *n* (%)**			1.654	0.161
Employment	81(36.3%)	9.92(3.45)		
Unemployment	41(18.4%)	10.54(3.64)		
Unemployed	77(34.5%)	10.53(3.75)		
Retirement	7(3.1%)	12.57(4.16)		
Others	17(7.6%)	9.17(3.78)		
**Smoking *n* (%)**			7.967	**0.005**
No	69(30.9%)	9.32(3.41)		
Yes	154(69.1%)	10.67(3.70)		
**Drinking *n* (%)**			8.448	**0.004**
No	43(19.3%)	9.05(3.98)		
Yes	180(80.7%)	10.59(3.48)		
**Taking drugs *n* (%)**			0.031	0.861
No	211(94.6%)	10.25(3.69)		
Yes	12(5.4%)	10.07(2.95)		
**ACEs**	**Yes**		**No**	
	N(%)		N(%)	
**Childhood maltreatment**				
Emotional neglect	56(21.2%)		208(78.8%)	
Physical neglect	183(69.3%)		81(30.7%)	
Emotional abuse	172(65.2%)		92(34.8%)	
Physical abuse	153(58.0%)		111(42.0%)	
Sexual abuse	158(59.8%)		106(40.2%)	
**Family/ household dysfunction**				
Living with substance abuser	26(9.8%)		238(90.2%)	
Living with household members who were mentally ill or suicidal	23(8.7%)		241(91.3%)	
Living with household member who were imprisoned	40(15.2%)		224(84.8%)	
Parental death, separation, or divorce	107(40.5%)		157(59.5%)	
Domestic violence	194(73.5%)		70(26.5%)	
**Violence outside the home**				
Bullying	135(51.1%)		129(48.9%)	
**IPV after age 18 years**				
Experienced psychological IPV	203(76.9%)		61(23.1%)	
Experienced physical IPV	176(66.7%)		88(33.3%)	
Experienced sexual IPV	159(60.2%)		105(39.8%)	
Experienced transgender-specific IPV	202(76.5%)		62(23.5%)	

IPV^a^: intimate partner violence coded as a dichotomous variable; IPV^b^: intimate partner violence coded as a continuous variable; SD: standard deviation; RMB: Renminbi; ACEs: adverse childhood experiences.

### Procedures

A snowball sampling approach was employed for recruitment, facilitated by a non-governmental organization (NGO). The NGO’s staff, catering to transgender women, diligently reached out to all their clients and conducted outreach activities for recruitment. With the support of the NGO, five qualified transgender women were selected as “seeds”. These “seeds” subsequently recruited or recommended additional suitable individuals, and all participants propagated the questionnaire survey in this manner until saturation was achieved (when no further participants could be introduced). The saturation was additionally verified by the NGO leaders, who collaborated closely with local transgender women and possessed a rough estimate of the transgender female population residing in the area. Before their participation, all individuals provided written informed consent, and subsequently, they distributed the questionnaire survey among others, continuing this process until they were unable to identify any further individuals who met the inclusion criteria. Once completing the survey, participants were requested to furnish demographic details, such as their age, sexual orientation, income, and other relevant information. Second, they completed the measures of ACEs, self-esteem, LGBT minority stress, and IPV. Note that the sub-scales are presented in the order shown to avoid potential reluctance or discomfort that could arise from addressing sensitive issues too early in the survey process. By beginning with less sensitive topics, participants may feel more at ease and be more likely to complete the survey, which in turn helps to ensure a higher response rate and more reliable data collection. Furthermore, logical presentation sequence can reflect the relationship among variables. Finally, upon submitting the questionnaire, participants received a 200 Chinese Yuan incentive, equivalent to approximately 30 U.S. dollars.

### Ethical approval

The ethical approval for the studies that engage human participants has been granted by the Ethics Committee at the School of Public Health, Shanghai Jiao Tong University, in China (Protocol # 2016022), which covers the Declaration of Helsinki requirements.

### Measures

#### Background characteristics

The following background information was recorded, including age, sexual orientation, educational attainment, marital status, monthly income, duration of living in Shenyang, working condition, smoking status, drinking status, and drug use. Age was analyzed as a continuous variable. Other variables were represented as categorical variables (Table 1).

#### Adverse childhood experiences

ACEs, assessed by Adverse Childhood Experiences International Questionnaire (ACE-IQ) [37,38], implement a 29-item assessment tool to evaluate exposure to three categories of childhood adversities, namely “childhood maltreatment”, “family/household dysfunctionality”, and “violence experienced outside the home”. Jointly, these domains embody 13 diverse classifications of ACEs. The domain of childhood maltreatment encompasses emotional neglect (2 items), physical neglect (3 items), emotional abuse (2 items), physical abuse (2 items), and sexual abuse (4 items). The domain of family/household dysfunctionality encompasses residing with a substance abuser (1 item); cohabiting with a household member experiencing mental illness or suicidal thoughts (1 item); sharing a household with an incarcerated member (1 item); undergoing parental death, separation, or divorce (2 items); and experiencing domestic violence (3 items). The domain of violence experienced outside the home encompasses bullying (1 item), observing community violence (3 items), and experiencing exposure to war or collective violence (4 items). Participants were invited to reflect on their experiences from the first 18 years of their lives and provide answers to the posed questions. The choices for answering each question could potentially fall into a dichotomous category (i.e. Yes/No, residing with a substance abuser; cohabiting with a household member experiencing mental illness or suicidal thoughts; sharing a household with an incarcerated member; undergoing parental death, separation, or divorce. A “Yes” response scores 1 point, while a “No” response scores 0 points). Based on a 5-point Likert scale, spanning from “Never” to “Always”, for the two items pertaining to emotional neglect, a response of “Never” to any of them earns 1 point, whereas all other responses are assigned 0 points, or based on a 4-point Likert scale, spanning from “Never” to “Many times”, for all eight remaining items. In consideration of the actual conditions, observing community violence and experiencing exposure to war or collective violence were excluded. The overall count of ACE categories that individuals encounter is aggregated to formulate an ACE score spanning from 0 to 11. The higher score indicates a greater number of ACEs. In summary, the ACE-IQ exhibited excellent internal consistency in the present research, yielding a Cronbach’s alpha score of 0.882.

#### Intimate partner violence

IPV, assessed by a five-item scale developed for the Transgender Youth Research Project [39], evaluates lifetime experiences of physical, psychological, sexual, and transgender-specific types of IPV by sexual partners. Response categories spanned from “1 = Never” to “4 = Multiple times”. When totaled the scores for the scale extended from 5 to 20, where a code of 1 signified agreement (ranging from 2 to 4) on any of the five items, and a code of 0 denoted a lack of experience with any form of IPV. The median score was 5 and the Cronbach’s was 0.858. Overall, the sample of the scale demonstrated good internal consistency in the present study, with a Cronbach’s alpha of 0.858.

#### Self-esteem

Self‐esteem levels were assessed utilizing the Chinese version of the Rosenberg Self‐Esteem Scale (RSES) [40]. Comprising 10 items, this scale gauges global self‐esteem based on positive and negative beliefs. Respondents evaluated each item utilizing a 4-point Likert scale, ranging from 0 (“Strongly disagree”) to 3 (“Strongly agree”), which ultimately produced a score from 0 (“Poor”) to 30 (“Excellent”). The scale employs reverse‐scoring for negatively worded items and is scored by summing the responses. The present study revealed a high reliability coefficient for the RSES, with a Cronbach’s α value of 0.741.

#### LGBT minority stress

Minority stress was assessed using the LGBT Minority Stress Measure [29], a 19-item self-report scale used to measure the sexual minority stress of LGBT, including five distinct constructs: community connectedness (5 items), internalized stigma (3 items), rejection anticipation (4 items), identity concealment (4 items), and victimization events (3 items). The items are scored on a 5-point Likert scale where respondents can express their agreement on a scale from 1 (“Strongly disagree”) to 5 (“Strongly agree”). The scoring process involves reversing the responses for the community connectedness items. A higher score on the scale reflects increased levels of minority stress. The overall scale demonstrated high internal consistency (Cronbach’s α = 0.718), the Cronbach’s α of community connectedness, internalized stigma, rejection anticipation, identity concealment, and victimization events were 0.872, 0.692, 0.645, 0.805, and 0.792, respectively.

### Statistical analysis

The statistical software SPSS, version 26 for Windows (IBM SPSS Inc.), was utilized to analyze the data, alongside the assistance of the PROCESS macro. Firstly, the reliability analysis of the questionnaires tailored specifically for this sample (in the Measures section) was conducted. Secondly, a comprehensive descriptive analysis, encompassing means and standard deviations as well as quantity and proportion, was undertaken. To obtain the prevalence of ACEs and IPV, we also estimated the two standard measures of ACEs and IPV (a continuous score and a dichotomous variable, 0 vs. any ACE; 0 vs. any IPV). Associated socio-demographic factors of IPV were explored by using ANOVA models. *P*-values that were less than 0.05 indicated statistical significance in the analysis. After conducting correlation analysis, correlation analysis was assessed with Pearson correlation test by controlling demographics (e.g. age, educational level, monthly income). PROCESS macro [41] was employed to ascertain the total, indirect effects, and conditional indirect effects. For the purpose of determining the statistical significance of the predicted sign associations, this study relied on 5,000 bootstrapping resamples to generate 95% confidence intervals (CIs) for two-tailed tests. The dependent variable was IPV, the independent variables were ACEs, self-esteem, and minority stress, and demographics (e.g. age, educational level, monthly income) were entered as control variables.

## Results

### Descriptive results

Table 1 provides a comprehensive overview of the demographic information and the key differences observed in the main variables. The study involved a total of 264 Chinese transgender women, the average age of the participants was 29.99 ± 6.38 years old, with a range of 18–53 years old. The ANOVA test revealed significant differences in the severity of IPV across various levels of monthly income, as well as among individuals with different smoking and drinking habits. In the final post hoc tests, we observed that individuals with a low monthly income (RMB 3,000 and below) exhibited significantly higher levels of IPV compared to those with a higher monthly income (RMB 6,001 and above; *ps* < 0.05). Additionally, transgender women who smoked or drank had a higher rate of IPV compared to those who did not engage in these habits (*ps* < 0.05). In terms of ACEs, the five most common ACEs were domestic violence (73.5%), physical neglect (69.3%), emotional abuse (65.2%), sexual abuse (59.8%), and physical abuse (58.0%). IPV survivors experienced various forms of IPV in their lifetime, including psychological IPV (76.9%), transgender-specific IPV (76.5%), physical IPV (66.7%), and sexual IPV (60.2%).

### Correlation results

Descriptive statistics and correlations among the variables were shown in Table 2: ACEs were found to be significantly inversely correlated with self-esteem (*r* = −0.363, *p* < 0.001), and positively associated with LGBT minority stress, and IPV (0.236 < *r* <  0.449, *ps* < 0.001). There were negative associations among self-esteem, LGBT minority stress and IPV (−0.401 < *r* < −0.334, *ps* < 0.001). LGBT minority stress had a positive relationship with IPV (*r* = 0.444, *p* < 0.001).

**Table 2.** Correlations among the study variables for transgender women.

Variables	1	2	3	4
1.ACEs	1			
2.Self-esteem	−0.363***	1		
3.LGBT minority stress	0.236***	−0.334***	1	
4.IPV	0.449***	−0.401***	0.444***	1
Mean	4.72	27.19	55.45	10.24
SD	2.62	4.36	8.34	3.65
Medium	5.00	26.00	57.00	11.00
IQR	5.00	5.00	8.00	6.00

ACEs: adverse childhood experiences; IPV: intimate partner violence; SD: standard deviation; IQR: inter quartile range.

*p < 0.05, **p < 0.01, *** *p* < 0.001.

### Mediation model testing

**Figure 1.**
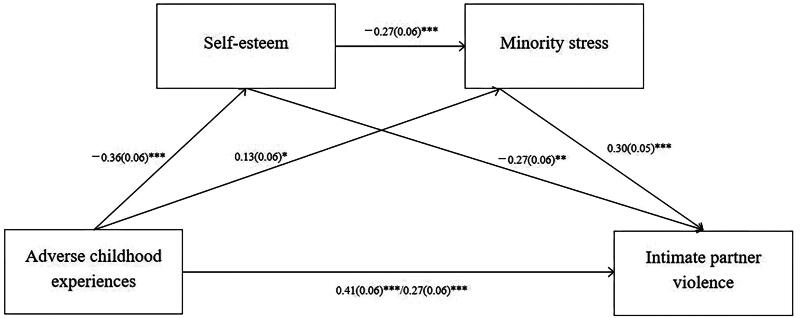
Effect model of adverse childhood experiences on intimate partner violence through self-esteem and minority stress. * *p* < 0.05, ** *p* < 0.01, *** *p* < 0.001.

Mediation analyses were performed *via* the bootstrap protocol, as recommended by Preacher and Hayes [41]. During the analysis process, 5,000 bootstrap samples were drawn, and the results were presented with bias-corrected 95% bootstrap confidence intervals (CI) for enhanced precision and clarity. Based on the research conducted by Preacher and Hayes [41], CIs that exclude zero suggest a noteworthy indirect influence of the independent variable (ACEs) on the dependent variable (IPV), mediated by self-esteem and LGBT minority stress, even when controlling for factors such as monthly income, smoking, and drinking habits. Results of the regression analyses for mediation are listed in Table 3 and Figure 1.

**Table 3.** Regression analyses for ACEs, self-esteem and LGBT minority stress in IPV.

Variables	Model 1: Self-esteem	Model 2: LGBT minority stress	Model 3: IPV	Model 4: IPV
ACEs	−0.36(0.06)***	0.13(0.06)*	0.41(0.06)***	0.27(0.06)***
Self-esteem		−0.27(0.06)***		−0.20(0.06)**
LGBT minority stress				0.30(0.05)***
*R* ^2^	0.16	0.14	0.24	0.38
*F*	12.55	8.34	20.25	26.33
*P*	< 0.001	< 0.001	< 0.001	< 0.001

ACEs: adverse childhood experiences; IPV: intimate partner violence. Monthly income, smoking, and drinking were controlled as covariates.

* *p* < 0.05, ** *p* < 0.01, *** *p* < 0.001.

The direct effect of ACEs on IPV was significant (the estimated effect*=* 0.4079, 95%CI: 0.2969–0.5189). Furthermore, the findings revealed two noteworthy indirect impacts of ACEs on IPV: through self-esteem (the estimated effect = 0.0708, 95%CI: 0.0152–0.1327), LGBT minority stress (the estimated effect = 0.0404, 95%CI: 0016–0.0847) or joint self-esteem and LGBT minority stress (the estimated effect = 0.0298, 95%CI: 0.0105–0.0591). After including self-esteem and LGBT minority stress as joint mediators, the direct effect of ACEs on IPV was still significant (the estimated effect = 0.2668, 95%CI: 0.1584–0.3753). In total, the standardized mediating effect size was 0.1411 (95%CI: 0.0700–0.2230), representing 34.59% of the total effect size (0.4079). Specifically, self-esteem contributed 17.36% (the indirect effect size of 0.0708), LGBT minority stress contributed 9.90% (the indirect effect size of 0.0404), and joint self-esteem and LGBT minority stress contributed 7.31% (the indirect effect size of 0.0298).

## Discussion

In this research, we aimed to explore whether ACEs can predict the occurrence of IPV in adulthood among Chinese transgender women. Furthermore, we investigated the potential mediating role of self-esteem and minority stress in these relationships. In the present study, transgender women’s IPV survivors experienced different forms of IPV in their lifetime, including experienced psychological IPV (76.9%), transgender-specific forms of IPV (76.5%), physical IPV (66.7%), and sexual IPV (60.2%), which were higher than the prevalence mentioned in previous literature in China [5,7,8,39]. The five most prevalence ACEs were domestic violence (73.5%), physical neglect (69.3%), emotional abuse (65.2%), sexual abuse (59.8%), and physical abuse (58.0%). In line with previous studies, childhood abuse and neglect emerged as the most frequently reported ACEs [13,42]. Thus, it can be seen, that ACEs and IPV in adulthood are prevalent among Chinese transgender women.

Consistent with previous research and theoretical frameworks, our findings indicate a notable correlational link between ACEs, self-esteem, minority stress, and IPV among transgender women, and ACEs can predict IPV in adulthood. The results were consistent with those of Zhu et al. [9]. These findings highlight the potential usefulness of trauma-informed care in the context of IPV screening, intervention, and prevention is essential, because individuals engaged in IPV may have a higher likelihood of having a background marked by exposure to ACEs. Self-esteem acts as a partial mediator in the relationship between ACEs and IPV in adulthood. This implies that ACEs may diminish self-esteem, subsequently reducing self-acceptance, thereby increasing the likelihood of experiencing IPV within intimate partnerships. The discoveries regarding the role of self-esteem as a mediator between childhood social environments and adult intimate relationships are congruent with the fundamental principles of attachment theory [43]. Furthermore, the sociometer theory postulates that self-esteem functions as a gauge, measuring one’s perceived value in relation to their interpersonal partners [44], with the belief of being liked and appreciated fostering an enhancement in self-esteem [45]. Thus, self-esteem can explain partially the association between ACEs and IPV in adulthood.

Our findings support initial hypotheses, showing that minority stress partially mediates ACEs-IPV connection in adulthood, similar to husbands’ results in previous research [30]. Childhood adversity affects IPV by increasing minority stress in transgender women. This could be due to lack of coping mechanisms, making them more prone to violence in intimate relationships [30]. It is the first study to explore the minority stress experienced by transgender women. Minority stress theory is the prevailing theory claiming that interpersonal victimization, internalized stigma, and rejection experienced by sexual minorities contribute to heightened levels of stress, potentially resulting in mental health and behavioral concerns [32,33]. Some researchers have proposed that integrating ACEs with minority stress theory can potentially clarify the causality sequence problem, given that ACEs precede the assessment of adults’ mental and behavioral health status. Furthermore, early encounters with adversity during childhood sensitize the brain, triggering its perception of the world as progressively more menacing and heightening one’s awareness to potential dangers within the environment [46]. In reality, contemporary research has established a clear correlation between exposure to ACEs and the perception of discrimination in adulthood [47,48]. Thus, it may be concluded that ACEs have the potential to intensify both the recognition and consciousness of facing distant minority stress encounters in adulthood, such as cis heterosexism. Additionally, they may heighten proximal minority stress processes, like the fear of rejection and identity concealment. Youth from sexual and gender minority communities, who have experienced a confluence of ACEs, are likely to be more acutely attuned to potential threats in their surroundings as a result of neurodevelopmental alterations stemming from trauma exposure [46], particularly concerning their minority identity. This, in turn, diminishes perceptions of social safety [49]. In addition, a noteworthy finding was that the joint self-esteem and minority stress mediated the effect of ACEs and IPV. Previous studies have revealed that individuals with lower self-esteem tend to respond to rejection by evaluating themselves in a more negative light, making them more susceptible of stressors due to transgender women’s identity, who were prone to IPV in an intimate relationship in adulthood. From a preventative viewpoint, the research highlights the cruciality for transgender women to identify ACEs, increase self-esteem, and decrease minority stress, and engage in considering the integrations between ACEs before 18 years old and IPV in adulthood [46].

The current study has several limitations that warrant acknowledgment. Firstly, as a cross-sectional study, it lacks the capacity to explore the causal relationships between IPV and risk factors in Chinese transgender women. Consequently, we cannot assert the existence of direct causal links among ACEs, self-esteem, minority stress, and IPV. Therefore, the primary findings of this study ought to be considered as preliminary in nature. Nevertheless, given the fact that ACEs took place prior to the age of 18 years, and that IPV was evaluated during adulthood, there existed a certain directionality in the observed relationships. Furthermore, the data were self-reported, potentially introducing desirability bias and recall bias. Secondly, the sample size is relatively small due to the concealment of transgender identity among transgender women. Utilizing snowball sampling methodology in Shenyang implies potential limitations in extrapolating the study’s findings to other regions within China. Lastly, the current study evaluates transgender-specific IPV, yet it remains unclear whether the participants were disclosing IPV experiences exclusive to their romantic relationships or encompassing a broader context. Consequently, future endeavors are imperative to scrutinize IPV through multiple and potentially more objective metrics.

## Conclusions

There exists a high prevalence of ACEs and IPV in adulthood among transgender women. Transgender women who have experienced ACEs also report a high prevalence of IPV in adulthood, both of which are associated with low self-esteem and high minority stress. Furthermore, the sequential mediation model demonstrates that self-esteem and minority stress have the potential to mediate the association between ACEs and IPV respectively, the combined mediating influence of self-esteem and minority stress can similarly exert such an effect. These findings have important implications for domestic violence service providers and other health professionals. Specifically, service providers should implement trauma-informed care approaches that address the long-term effects of ACEs, and create IPV prevention programs that actively support self-esteem and offer coping mechanisms for minority stress. Additionally, healthcare professionals need to be trained to recognize the compounded vulnerabilities faced by transgender women with a history of ACEs, allowing them to provide more targeted and empathetic care. Future research ought to persist in investigating IPV within the transgender community, striving to unravel the underlying risk factors for IPV while also identifying protective factors, with the aim of fostering health equity for this vulnerable population.

## Data Availability

The findings of this study are supported by data that can be acquired upon request by contacting the corresponding author. Nevertheless, the data cannot be publicly disclosed owing to privacy and ethical considerations.
